# Supplementary material for: Genetic variants associated with platelet count are predictive of human disease and physiological markers
Source: Commun Biol. 2021 Sep 27;4:1132. doi: 10.1038/s42003-021-02642-9 (PMC8476563; doi:10.1038/s42003-021-02642-9)
Supplement: Supplementary file 2 — Supplementary Information [file 42003_2021_2642_MOESM2_ESM.pdf]

Genetic variants associated with platelet count are predictive of human disease and physiological markers

Evgenia Mikaelsdottir, Gudmar Thorleifsson, Lilja Stefansdottir, Gisli Halldorsson, Jon K. Sigurdsson, Sigrun H. Lund, Vinicius Tragante, Pall Melsted, Solvi Rognvaldsson, Kristjan Norland, Anna Helgadottir, Magnus K. Magnusson, Gunnar B. Ragnarsson, Sigurdur Y. Kristinsson, Sigrun Reykdal, Brynjar Vidarsson, Ingibjorg J. Gudmundsdottir, Isleifur Olafsson, Pall T. Onundarson, Olof Sigurdardottir, Emil L. Sigurdsson, Gerdur Grondal, Arni J. Geirsson, Gudmundur Geirsson, Julius Gudmundsson, Hilma Holm, Saedis Saevarsdottir, Ingileif Jonsdottir, Gudmundur Thorgeirsson, Daniel F. Gudbjartsson, Unnur Thorsteinsdottir, Thorunn Rafnar, Kari Stefansson

Additional Information

Following Supplementary Material is available for this paper.

- Supplementary Figures 1-4 (available in this file)
- Supplementary Data 1-25 (available as separate MS Excel files)

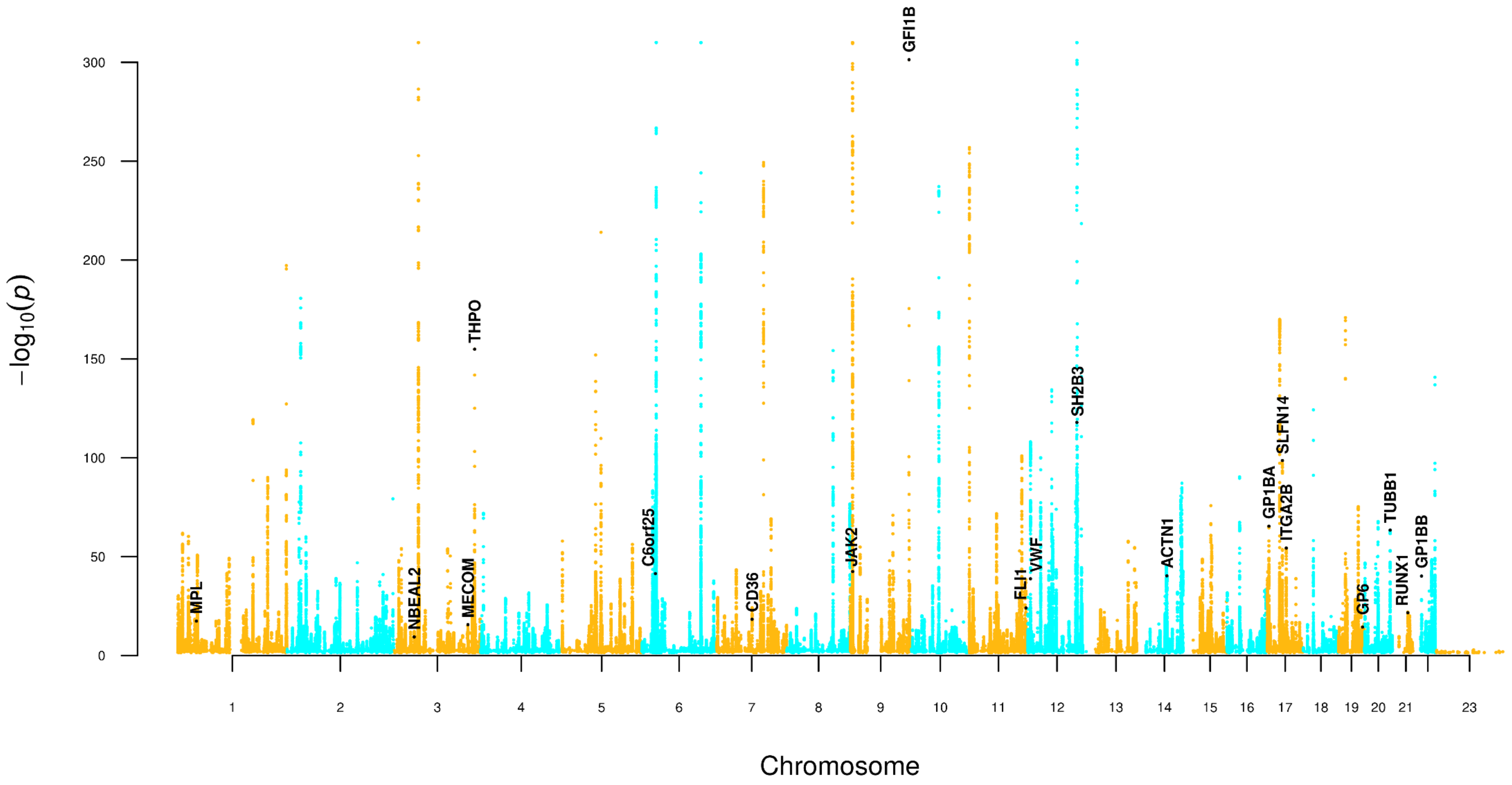

Supplementary Figure 1. Manhattan plot. The plot shows all variants in the meta-analysis that associate with PLT with p-value ≤ 0.05, are present in both populations, have info > 0.8 in Iceland and > 0.7 in the UK and no quality issues. Presented are negative $\log_{10}$-transformed p-values from an inverse variance weighted meta-analysis of association results from Iceland and UKB, n=536,974 (y-axis) over the 23 chromosomes (x-axis). Names of genes, which are implicated in platelet-related disorders and harbor genome-wide significant variants, are marked with black. For details on associations, see **the GWAS Summary Statistics**.

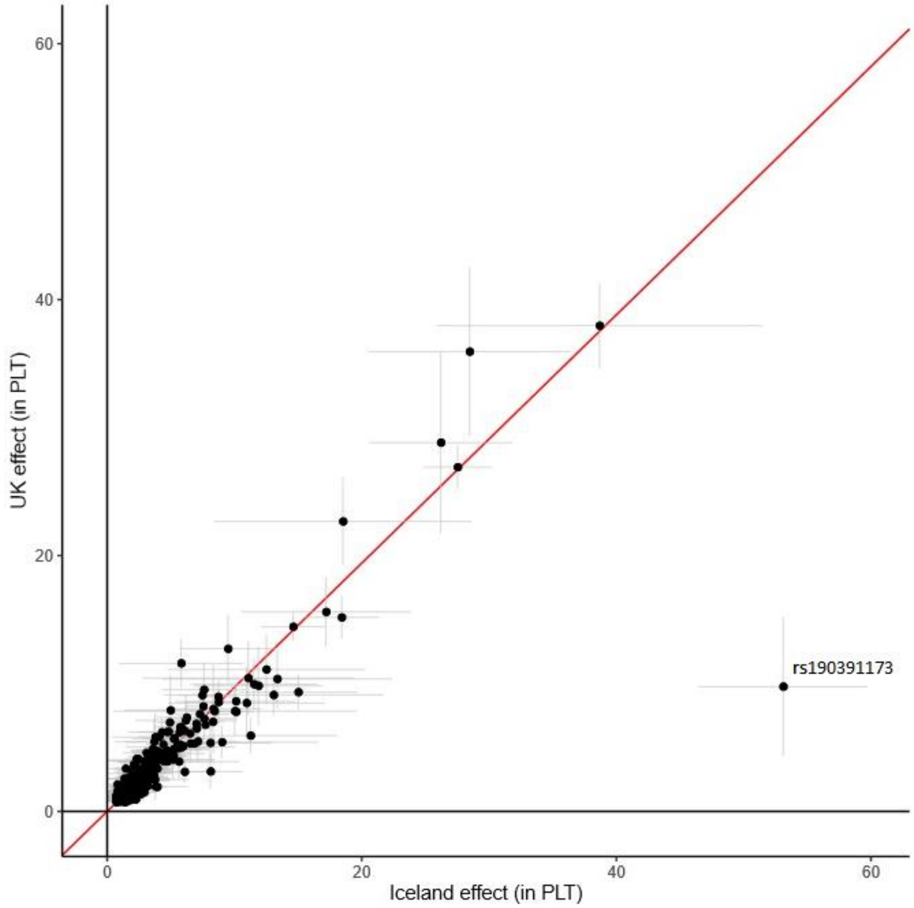

Supplementary Figure 2. Effects of the 577 PLT variants in Iceland vs. the UK. Shown are effects of the 577 PLT variants expressed as platelet count (PLT, in N×10^3/µl) with 95% confidence intervals, in Iceland vs the UK. The outlier, rs190391173, is a very rare variant in the UK (minor allele frequency (MAF) = 0.07%) with imputation information 0.88, whereas it is slightly more frequent (MAF=0.26%) and much better imputed in Iceland (imputation information =1), which may explain the observed effect difference. For detailed information on associations, see **Supplementary Data 2.**

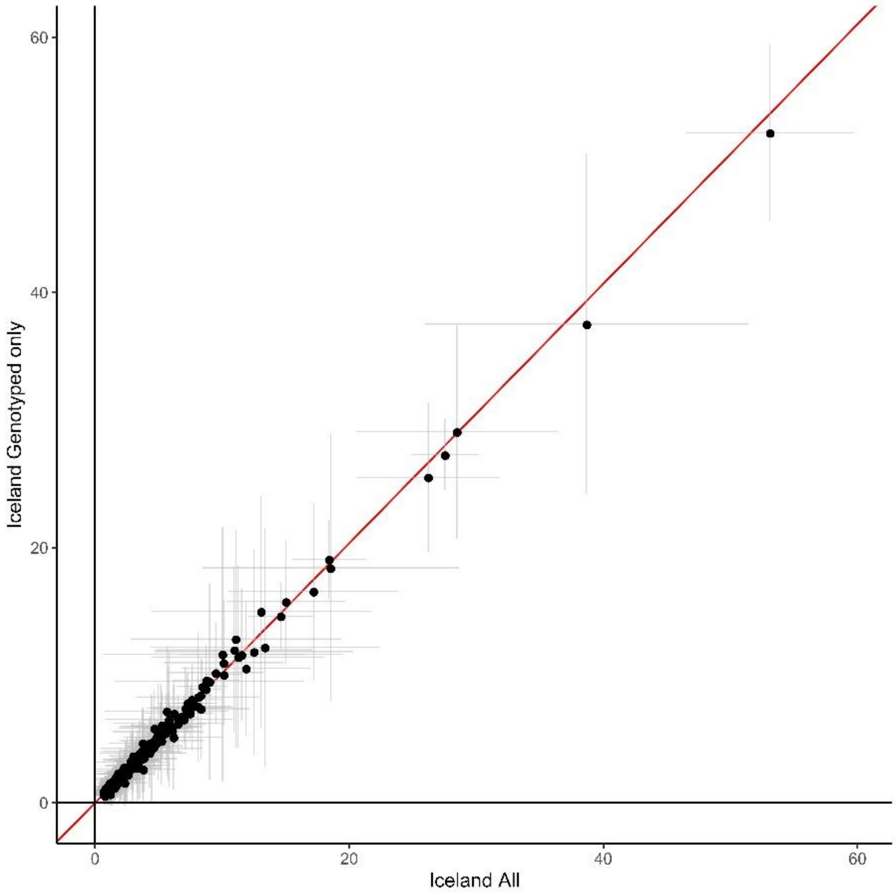

Supplementary Figure 3. Comparison of data including both genotyped and familially imputed Icelanders vs. only genotyped Icelanders. The plot shows effects of the 577 PLT variants expressed as platelet count (PLT, in N×10^3/µl) with 95% confidence intervals, in data including both genotyped and familially imputed Icelanders (x-axis) vs. only genotyped Icelanders (y-axis). For detailed information, see **Supplementary Data 23**.

a

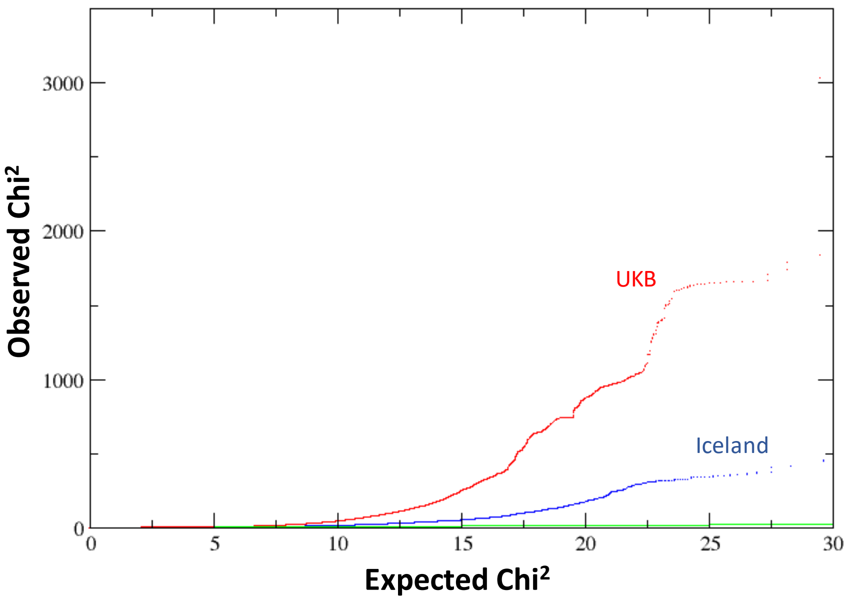

b

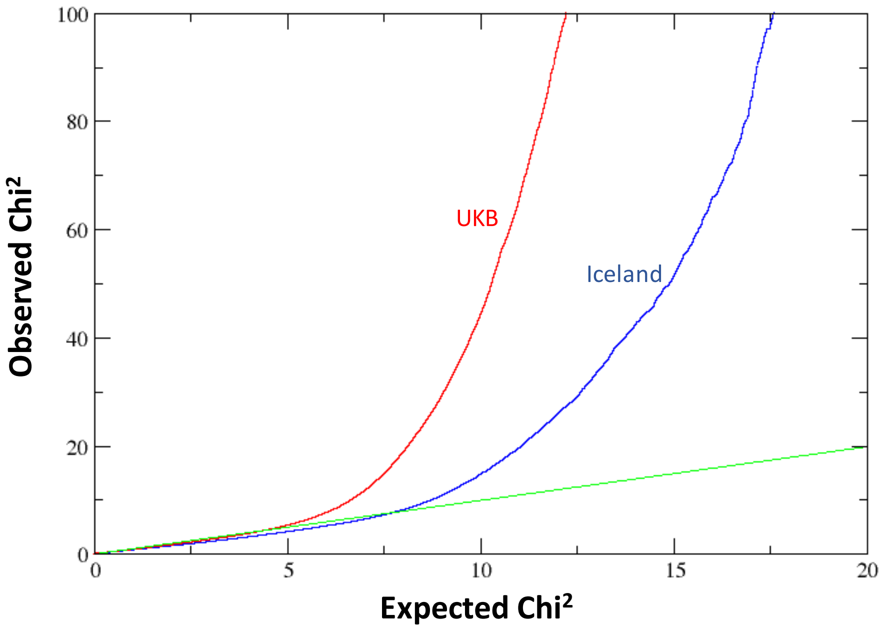

Supplementary Figure 4. Q-Q plots of the adjusted Chi^2–statistics from a linear regression of PLT values on allele counts for all tested variants**.** (a) A Q-Q plot of the adjusted Chi^2–statistics for association with PLT values for all tested variants for the UK Biobank (n=397,495, red dots) and the Icelandic (n=139,479, blue dots) datasets, respectively. The equiangular line (green line) is included in the plot for reference purpose. (b) A subset of (a) showing the Q-Q plot for variants with low Chi^2 values.
