# Supplementary material for: Genetic variants associated with platelet count are predictive of human disease and physiological markers
Source: Commun Biol. 2021 Sep 27;4:1132. doi: 10.1038/s42003-021-02642-9 (PMC8476563; doi:10.1038/s42003-021-02642-9)
Supplement: Supplementary file 3 — Description of Additional Supplementary Files [file 42003_2021_2642_MOESM3_ESM.pdf]

Description of Additional Supplementary Files

File name: Supplementary Data 1-25

Description:

Captions for each Supplementary Data item are as follows:

Supplementary Data 1. Variant types, their expected impact and number of variants in each impact class (summarized (Meta) and individually for each cohort (Iceland and UK)) and class-specific genome-wide significance thresholds applied in the meta-analysis. Variants included in the analysis: imputation information > 0.8 and minor allele frequency (MAF) > 0.01% (Iceland) and imputation information > 0.7 and MAF > 0.01% (UK). Symbols and abbreviations: DHS, DNase hypersensitivity sites; P threshold, meta p-value thresholds for genome-wide significance of variants in different annotations classes (see Methods).

Supplementary Data 2. The 577 variants reaching genome-wide significance in meta-analysis of PLT. Effect sizes are shown with respect to the effector allele and expressed in number of platelets (in 103/μl) (see Methods). Symbols and abbreviations: EA, effector allele; EAF% Ice/UK, effector allele frequency in Iceland and the UK in %; P, p-value; Phet, p-value for heterogeneity in the effect estimate between the Icelandic and UK Biobank data; P adj and Effect adj (95% CI), adjusted meta p-value and effect after conditioning on other significant variants in the tested window (+/- 10Mb); NA, the variant represents the only significant signal in the tested window; * Variant in or near a gene implicated in a platelet-related disorder according to the Online Mendelian Inheritance in Man database (OMIM, www.omim.org) (see also Supplementary Data 8 and 9) ; § Variant located ≥ 1Mb away from the nearest known PLT variant reported in the GWAS catalog as of May 26, 2021; ł Associations presented in Fig. 3; ǂ Secondary signal.

Supplementary Data 3. The Icelandic and UK cohorts representing the discovery trait, platelet count (PLT), and phenotypes significantly correlated with the platelet count polygenic risk score (PLT PRS). Combined: Total number of directly and familially imputed individuals available for association analysis in Iceland.

Supplementary Data 4. Platelet count polygenic risk score and correlation with the tested hematologic diseases. The applied significance threshold was 1×10-5 (see Methods). Symbols and abbreviations: nAff, number of cases; nCon, number of controls; P, p-value.

Supplementary Data 5. Association of the PLT PRS with the 25 diseases and traits after exclusion of the MHC region from the polygenic risk score calculations. Abbreviations: AP, alkaline phosphatase; AS, Ankylosing spondylitis; BPH, benign prostate hyperplasia; CRP, C-reactive protein; E, effect; GGTP, gamma glutamyl transpeptidase; MPN, myeloproliferative neoplasm; MPV, mean platelet volume; P, p-value; Phet, p-value for heterogeneity in the effect estimate between the Icelandic and UK Biobank data; PLT, platelet count; RA, rheumatoid arthritis; WBC, white blood cell count.

Supplementary Data 6. PLT variants and association with the tested quantitative traits (QTs). Only significant associations are presented. The applied significance threshold was combined p-value ≤ 3.94 x 10-6. Effects are expressed in standard deviations (STD) and presented with respect to the PLT increasing allele for which the effect is also presented in STD (PLT Emeta (in STD) column). Symbols and abbreviations: E, effect; EA, effector allele that is also the PLT increasing allele; OA, other allele; P, p-value; ǂ Variant associated with the tested diseases (Supplementary Data 7); § Association with mean platelet volume with the effect direction opposite to predictions of the PLT PRS analysis (see Table 1).

Supplementary Data 7. Association of the PLT variants with the diseases defined in the PLT PRS analysis. Only significant associations are shown. Symbols and abbreviations: * Associations with relevant phenotypes reported for the variant identified in this study or correlated ones that represent the same signal based on results of conditional analyses. BP, blood pressure; DBP, diastolic blood pressure; HT, hypertension; MAP, mean arterial pressure; MetS, metabolic syndrome; MPN, myeloproliferative neoplasm; NA, not applicable; RA, rheumatoid arthritis; SBP, systolic blood pressure; TG, triglycerides; x, interaction/pairwise combination of traits.

Supplementary Data 8. Genes involved in platelet disorders that harbor the identified PLT variants. Platelet disorders were defined based on information from the Online Mendelian Inheritance in Man database (OMIM, www.omim.org) and confirmed through literature. Symbols and abbreviations: AD, autosomal dominant; AR, autosomal recessive; SMu, somatic mutation; OMIM #, a phenotype number in the Online Mendelian Inheritance in Man catalog (see https://www.omim.org/); MoI, mode of inheritance; * Coding sequence variant(s) in the gene found to associate with PLT in this meta-analysis.

Supplementary Data 9. Index variants of lower impact classes and their correlated high or moderate impact class variants (see Supplementary Data 1 for impact class definition). Neither variant remains significant after conditioning on the other one (Supplementary Data 10). Symbols and abbreviations: EA, effector allele; OA, other allele; * Coding sequence variant in a gene implicated in a platelet-related disorder according to the Online Mendelian Inheritance in Man database (OMIM, www.omim.org) (see Supplementary Data 8).

Supplementary Data 10. Results of conditional analyses for the index SNPs and their correlated coding sequence variants listed in Supplementary Data 9. Symbols and abbreviations: Effect, effect expressed in number of platelets (in 103/ml); P, p-value; P adj and Effect adj, p-value and effect after conditioning on the correlated variant (Conditioned on); NA, P adj is reaching 1 and Effect adj is reaching 0; Ref A, reference allele.

Supplementary Data 11. Genes not expressed in platelets or megakaryocytes and harboring coding PLT variants with functions in cholesterol/lipid homeostasis and non-alcoholic fatty liver disease.

Supplementary Data 12. Enriched tissues and cell types identified by DEPICT. Significance criteria: False discovery rate (FDR) < 0.05.

Supplementary Data 13. Results of the gene set enrichment analysis with DEPICT. Only significant results are presented. Significance criteria: p-value < 3.46E-06 (0.05 corrected with 14,461, the number of tested gene sets); § Gene sets presented in Fig.2.

Supplementary Data 14. Pearson correlations for the gene sets presented in Fig.2.

Supplementary Data 15. cis-eQTLs from all tissues that were defined as significantly enriched in the analyses with DEPICT. See Supplementary Data 12 for details on significantly enriched tissues, cell types and physiological systems. Symbols and abbreviations: Eexpr, effect on gene expression; EPLT, effect on PLT expressed in standard deviation (as in Fig. 3); EA, Effector allele; OA, Other allele, Pexpr, p-value referring to the expression data; PPLT, p-value referring to the PLT data; P Threshold, p-value threshold as defined in the GTEx datasets for the particular cis-eQTL; * Shown are expression data for a highly correlated SNP reported in the megakaryocyte and platelet cis-eQTL datasets, publicly available data from a recent study (PMID: 33094331). The p-value thresholds applied in analysis of the Icelandic data were ≤ 1.52×10-6 for blood and p-value ≤ 8.8×10-7 for the adipose tissue. For eQTLs identified in the platelet and megakaryocyte data, the significance threshold was set at the calculated q-value ≤ 0.05. See Methods for details.

Supplementary Data 16. Genes potentially involved in regulation of PLT based on results of this study. Symbols and abbreviations: eQTL, the gene is defined in the cis-eQTL analysis (Supplementary Data 15); mslof, the gene is identified in the coding variant analysis (Supplementary Data 2 and 9-10); * Known platelet gene implicated in a platelet-related disorder according to the Online Mendelian Inheritance in Man database (OMIM, www.omim.org) (Supplementary Data 8); § Gene expressed in megakaryocytes (PMID: 33094331); ł Gene expressed in platelets (PMID: 33094331); ǂ Gene expressed in platelets (PMID: 24524654).

Supplementary Data 17. Top 5 disease categories, cellular functions and physiological systems defined by the Ingenuity Pathway Analysis (IPA) based on the gene list that incorporates candidate genes identified in the cis-eQTL and coding variant analyses (Supplementary Data 16 and Fig.4). Symbols and abbreviations: * Platelet disorders; § Platelet development, morphology, and function; ǂ Myeloproliferative neoplasms.

Supplementary Data 18. Endogenous master regulators with Benjamini-Hochberg corrected p-value ≤ 0.05 identified by the IPA Core Analysis. Symbols and abbreviations: B-H P, Benjamini-Hochberg corrected p-value; Master Regulator, the predicted upstream regulator that orchestrates the causal network; P, p-value; Participating Regulators, regulators through which the upstream regulator molecule controls the expression of target molecules in the dataset; Target Molecules, molecules in the dataset whose expression is potentially controlled by the upstream regulator; Z, activation z-score predicting the activation state of the regulator; § Significantly activated or inhibited master regulators, for details on their regulated molecules, canonical pathways and diseases, see Supplementary Data 19.

Supplementary Data 19. Master regulators with the absolute values of the activation z-score ≥ 2 and Benjamini-Hochberg corrected p-value ≤ 0.05 identified by the IPA Core Analysis (Supplementary Data 18). The master regulators are presented along with their regulated canonical pathways and diseases, binding partners, regulators, and regulated proteins. Data source: the IPA's Ingenuity Knowledge Base. Italic bold black: Diseases and conditions associated with PLT and the PLT PRS (Table 1). Italic bold red: Canonical pathways in control of blood pressure. Italic bold orange: Canonical pathways in rheumatoid arthritis. Italic bold purple: Canonical pathways in regulation of PLT and platelet functions.

Supplementary Data 20. Effect of the PLT genes on toxicological functions as predicted by the IPA Core Analysis (see Methods). Significance criteria: Benjamini-Hochberg corrected p-value ≤ 0.05. Symbols and abbreviations: B-H P, Benjamini-Hochberg corrected p-value; P, p-value; NA, no prediction of activation z-score; Z, activation z-score.

Supplementary Data 21. Changes in canonical pathways identified by the IPA Core Analysis. Significance criteria: Benjamini-Hochberg corrected p-value ≤ 0.05. Symbols and abbreviations: P, p-value; B-H P, Benjamini-Hochberg corrected p-values; Z, activation z-score (see Methods); NA, no prediction of activation z-score. * Canonical pathways relevant to the diseases associated with the PLT PRS (Table 1) and regulated by the master regulator with significantly changed activity (Supplementary Data 19). § Canonical pathway meeting the significance criteria. For details on molecular mechanisms, diseases and functions regulated by the pathway, see Supplementary Data 22.

Supplementary Data 22. Molecular mechanisms, diseases and functions regulated by the significantly altered canonical pathways (Benjamini-Hochberg corrected p-value ≤ 0.05, see Supplementary Data 21). Data source: the IPA's Ingenuity Knowledge Base. Italic bold red: Molecular mechanisms in control of blood pressure. Italic bold blue: Molecular mechanisms of myelopoiesis and myeloid cell functions. Italic bold orange: Regulation of inflammatory response and autoimmunity. Italic bold purple: Platelet functions and known genes in monogenic platelet disorders.

Supplementary Data 23. Comparison of data including all Icelanders and data including genotyped Icelanders only. Effect sizes are shown with respect to the effector allele and expressed in number of platelets (in 103/μl) (see Methods). Symbols and abbreviations: Combined, data include both genotyped and familially imputed Icelanders; EA, effector allele; EAF% effector allele frequency in Iceland in %; Genotyped, data include genotyped Icelanders only; P, p-value; OA, other allele. See also Supplementary Figure 3.

Supplementary Data 24. Correction factors for the phenotypes identified as significant in the PLT PRS analyses.

Supplementary Data 25. Fraction of variance of PLT measurements explained by PLT-PRS scores created based on different re-weighting models in LDpred. Symbols and abbreviations: *Assumed fraction of causal variants in the LDpred model used.
