# Supplementary material for: Genetic variants associated with platelet count are predictive of human disease and physiological markers
Source: Commun Biol. 2021 Sep 27;4:1132. doi: 10.1038/s42003-021-02642-9 (PMC8476563; doi:10.1038/s42003-021-02642-9)
Supplement: Supplementary file 5 — Reporting Summary [file 42003_2021_2642_MOESM5_ESM.pdf]

nature research

Corresponding author(s): Evgenia Mikaelsdottir
Kari Stefansson

Last updated by author(s): Sep 2, 2021

Reporting Summary

Nature Research wishes to improve the reproducibility of the work that we publish. This form provides structure for consistency and transparency in reporting. For further information on Nature Research policies, see our Editorial Policies and the Editorial Policy Checklist.

Statistics

For all statistical analyses, confirm that the following items are present in the figure legend, table legend, main text, or Methods section.

n/a	Confirmed	
☐	☒	The exact sample size (n) for each experimental group/condition, given as a discrete number and unit of measurement
☒	☐	A statement on whether measurements were taken from distinct samples or whether the same sample was measured repeatedly
☐	☒	The statistical test(s) used AND whether they are one- or two-sided<br>*Only common tests should be described solely by name; describe more complex techniques in the Methods section.*
☐	☒	A description of all covariates tested
☐	☒	A description of any assumptions or corrections, such as tests of normality and adjustment for multiple comparisons
☐	☒	A full description of the statistical parameters including central tendency (e.g. means) or other basic estimates (e.g. regression coefficient) AND variation (e.g. standard deviation) or associated estimates of uncertainty (e.g. confidence intervals)
☐	☒	For null hypothesis testing, the test statistic (e.g. F, t, r) with confidence intervals, effect sizes, degrees of freedom and P value noted<br>*Give P values as exact values whenever suitable.*
☒	☐	For Bayesian analysis, information on the choice of priors and Markov chain Monte Carlo settings
☒	☐	For hierarchical and complex designs, identification of the appropriate level for tests and full reporting of outcomes
☐	☒	Estimates of effect sizes (e.g. Cohen's d, Pearson's r), indicating how they were calculated

Our web collection on statistics for biologists contains articles on many of the points above.

Software and code

Policy information about availability of computer code

Data collection	No specific computer code was used for data collection.
Data analysis	We used publicly available software (URLs listed below) in conjunction with the algorithms in the sequencing processing pipeline (Whole-genome sequencing, Association testing, RNA-seq mapping and analysis) as described in the Methods of our manuscript.<br>URLs:<br>Bedtools v2.25.0-76-g5e7c696z, https://github.com/arq5x/bedtools2/<br>BOLT-LMM, https://data.broadinstitute.org/alkesgroup/BOLT-LMM/downloads/<br>BWA 0.7.10 mem, https://github.com/lh3/bwa<br>Cytoscape, https://cytoscape.org/<br>DEPICT, https://data.broadinstitute.org/mpg/depict/<br>GenomeAnalysisTKLite 2.3.9, https://github.com/broadgsa/gatk/<br>Genome browser, https://genome.ucsc.edu/<br>GTEx, https://www.gtexportal.org/home/<br>GWAS catalog, https://www.ebi.ac.uk/gwas/<br>LD link, https://ldlink.nci.nih.gov/?tab=ldpair<br>LDSC (LD Score), https://github.com/bulik/ldsc<br>OMIM, https://www.omim.org/<br>Picard tools 1.117, https://broadinstitute.github.io/picard/<br>Platelet and megakaryocyte cis-eQTLs, http://www.biostat.jhsph.edu/~kkammers/GeneSTAR/<br>SAMtools 1.3, http://samtools.github.io/<br>Variant Effect Predictor, https://github.com/Ensembl/ensembl-vep

Ingenuity Pathway Analysis (IPA) was performed using commercially available software from QIAGEN Inc. (https://www.qiagenbioinformatics.com/products/ ingenuitypathway-analysis).

We used R extensively to analyze data and create plots.

No custom codes were created for this project.

For manuscripts utilizing custom algorithms or software that are central to the research but not yet described in published literature, software must be made available to editors and reviewers. We strongly encourage code deposition in a community repository (e.g. GitHub). See the Nature Research guidelines for submitting code & software for further information.

Data

Policy information about availability of data

All manuscripts must include a data availability statement. This statement should provide the following information, where applicable:

- Accession codes, unique identifiers, or web links for publicly available datasets
- A list of figures that have associated raw data
- A description of any restrictions on data availability

The sequence variants from the Icelandic population whole-genome sequence data have been deposited at the European Variant Archive under accession code PRJEB15197. The GWAS summary statistics and data on the polygenic risk score will be made available at https://www.decode.com/summarydata at the time of publication. The UK Biobank data can be obtained upon application (https://www.ukbiobank.ac.uk/). The authors declare that the data supporting the findings of this study are available within the article, its Supplementary Information file, and upon reasonable request.
Data presented in Fig.1, showing the overview of association of the PLT variants with other quantitative traits with respect to the PLT increasing allele, are provided in Supplementary Data 6.
Data presented in Fig. 2, which shows a network of gene sets identified in the DEPICT analyses, are provided in Supplementary Data 13 and 14.
Data points presented in Fig. 3, showing the PLT variants that affect gene expression, are provided in Supplementary Data 15 and Supplementary Data 2 Note ƚ.
Data points presented in Fig. 4 that shows association of the 284 candidate causal PLT genes with diseases, molecular functions, and physiologic systems are provided in Supplementary Data 17.
Data presented in Supplementary Figure 1, which shows the Manhattan plot, will be provided in the GWAS Summary Statistics.
Data points presented in Supplementary Figure 2, which shows effects of the 577 PLT variants in Iceland vs. the UK, are provided in Supplementary Data 2.
Data points presented in Supplementary Figure 3, which compares effects of the 577 PLT variants in data including both genotyped and familially imputed Icelanders and genotyped only Icelanders, are provided in Supplementary Data 23.

Field-specific reporting

Please select the one below that is the best fit for your research. If you are not sure, read the appropriate sections before making your selection.

☒ Life sciences ☐ Behavioural & social sciences ☐ Ecological, evolutionary & environmental sciences

For a reference copy of the document with all sections, see nature.com/documents/nr-reporting-summary-flat.pdf

Life sciences study design

All studies must disclose on these points even when the disclosure is negative.

Sample size	PLT GWAS included 536,974 Europeans from Iceland and the UK. Details on the cohorts (PLT and other traits tested) are provided in the Methods. No sample size calculation was performed as all available individuals were used for the study.
Data exclusions	No data were excluded.
Replication	We performed GWAS studies in two independent populations and combined the results. We present results for the populations independently and combined, and success of replication is reported on per-variant basis, both as individual p-values and as heterogeneity of effects between groups.
Randomization	No randomization was used and is not relevant within the context of a genome-wide association study.
Blinding	Not relevant for a genome-wide association study.

Reporting for specific materials, systems and methods

We require information from authors about some types of materials, experimental systems and methods used in many studies. Here, indicate whether each material, system or method listed is relevant to your study. If you are not sure if a list item applies to your research, read the appropriate section before selecting a response.

Materials & experimental systems

n/a	Involved in the study
☒	☐ Antibodies
☒	☐ Eukaryotic cell lines
☒	☐ Palaeontology and archaeology
☒	☐ Animals and other organisms
☐	☒ Human research participants
☒	☐ Clinical data
☒	☐ Dual use research of concern

Methods

n/a	Involved in the study
☒	☐ ChIP-seq
☒	☐ Flow cytometry
☒	☐ MRI-based neuroimaging

Human research participants

Policy information about studies involving human research participants

Population characteristics	The Icelandic study included 139,479 chip-typed individuals and their 130,732 familially imputed 1st and 2nd degree relatives with available PLT measurements. PLT measurements used in GWAS were obtained from 3 health care centers: the National University Hospital of Iceland (LSH), the Icelandic Medical Center (Laeknasetrid) Laboratory in Mjodd (RAM) in Reykjavik, Iceland, and Akureyri Hospital (SAK) in Akureyri, Iceland. The data included all measurements made in these laboratories in the period from 1993 to 2015. The year of birth ranged from 1893 to 2015 (median year of birth 1969), and 52.2% were females. Mean PLT ± standard deviation (SD) was 253,900/µl ± 101,200/µl, with average 12.7 measurements per person.<br>The UK Biobank dataset included PLT data for 397,495 chip-typed individuals. Mean PLT ± SD was 252,400/µl ± 59,900/µl, with 1.05 measurements per individual. These individuals are participants in a large prospective cohort study of ~500,000 volunteer participants, who were recruited between the ages of 40 and 69 years in 2006-2010 across the UK.
Recruitment	All individuals with PLT measurements, available genotypes and written informed consent.
Ethics oversight	The Icelandic National Bioethics Committee approved the study (reference number: VSN-15-023), including the protocol, methodology and all documents presented to the participants. All individuals who donated samples provided written informed consent. All sample identifiers were encrypted in accordance with the regulations of the Icelandic Data Protection Authority. Personal identities of the participants and biological samples were encrypted by a third-party system approved and monitored by the Icelandic Data Protection Authority.<br>All UK participants gave informed consent, and the UK Biobank's scientific protocol and operational procedures were reviewed and approved by the North West Research Ethics Committee (REC reference number 06/MRE08/65). This research has been conducted using the UK Biobank Resource under application numbers 24711 and 24898.

Note that full information on the approval of the study protocol must also be provided in the manuscript.
